# Sinonasal B‐cell lymphomas: A nationwide cohort study, with an emphasis on the prognosis and the recurrence pattern of primary diffuse large B‐cell lymphoma

**DOI:** 10.1002/hon.2968

**Published:** 2022-02-06

**Authors:** Patrick R. G. Eriksen, Erik Clasen‐Linde, Peter de Nully Brown, Laura Haunstrup, Mette Christoffersen, Peter Asdahl, Troels Møller Thomsen, Cecilie Dupont Harwood, Steffen Heegaard, Christian von Buchwald

**Affiliations:** ^1^ Department of Otorhinolaryngology, Head and Neck Surgery & Audiology Rigshospitalet Copenhagen Denmark; ^2^ Hematopathology Section, Department of Pathology Rigshospitalet Kobenhavn Denmark; ^3^ Department of Hematology Rigshospitalet University of Copenhagen Kobenhavn Denmark; ^4^ Department of Hematology Aalborg University Hospital Aalborg Denmark; ^5^ Department of Hematology Odense University Hospital Odense Denmark; ^6^ Department of Hematology Aarhus University Hospital Aarhus Denmark; ^7^ Department of Hematology Holstebro Central Hospital Holstebro Denmark; ^8^ Eye Pathology Section, Department of Pathology Rigshospitalet University of Copenhagen Kobenhavn Denmark

**Keywords:** lymphoma, nose neoplasms, paranasal neoplasms, prognosis, treatment

## Abstract

Lymphomas of the nasal cavity and paranasal sinuses (NPS) are rare. Knowledge on sinonasal B‐cell lymphoma (SNBCL) primarily comes from case series or single‐center studies on small cohorts. We sought to determine the subtype distribution, clinical characteristics, disease behavior, and prognosis on a nationwide scale, with an emphasis on prognostic factors for the most common sinonasal lymphoma, primary sinonasal diffuse large B‐cell lymphoma (PSDLBCL). We collated all data from medical records and national databases on patients registered with SNBCL from 1980 through 2018 in the national pathology registry and collected all tissue samples for validation of diagnosis. We included 205 patients and found 10 different subtypes of lymphoma. Diffuse large B‐cell lymphoma (DLBCL) was the predominant subtype (80%). The incidence of SNBCL was 0.14/100,000 person‐years. The five‐year progression‐free survival (PFS) and overall survival rates for PSDLBCL were 50% and 56%, respectively. For PSDLBCL, Rituximab showed a statistically significant effect (Hazard Ratio 0.22, *p < *0.001), whereas consolidative radiotherapy combined with immunochemotherapy was of limited value (PFS, *p = *0.93). When treatment failure occurred, DLBCL showed a distinct pattern of recurrence/dissemination to the NPS, skin, breast, central nervous system (CNS), and/or testis. Collectively, DLBCL comprised a clear majority of SNBCLs, although nine other subtypes were represented. Data showed that immunochemotherapy increased survival for PSDLBCL and that the addition of radiotherapy did not benefit patients. Furthermore, treatment failure for sinonasal DLBCL showed a possible common pathogenesis with primary extranodal lymphomas of specific locations (e.g., CNS, skin, breast, and testis).

## 1 INTRODUCTION

Lymphomas involving the nasal cavity and paranasal sinuses (the NPS), also known as sinonasal lymphomas, are rare extranodal lesions, which are sparsely characterized in the literature.

Hematolymphoid neoplasms account for around 14% of all sinonasal malignancies in Denmark, second only to squamous cell carcinoma, which accounts for almost 50%.^1^ It is estimated that 42%–94% of all sinonasal B‐cell lymphomas (SNBCLs) in Caucasian populations are of the subtype diffuse large B‐cell lymphoma (DLBCL).^2^, ^3^, ^4^, ^5^ The difference in reported rates is presumably due to the nature of earlier publications, in which SNBCL is described through case series, single‐center studies with small sample sizes, or registry studies without validation of diagnoses and location.

Diffuse large B‐cell lymphoma is classified as an aggressive lymphoma exhibiting a broad variation in pathobiological and clinical findings defined by the primary site of the lymphoma.^6^, ^7^, ^8^, ^9^ The disease behavior in extranodal DLBCLs has been linked to mutational patterns possibly acquired through somatic hypermutation. This discovery has provided a new understanding of the role of the primary site, especially in central nervous system (CNS), testicular, breast, cutaneous, and retinal DLBCL.^10^ This may also be the case for sinonasal DLBCL, as older and smaller studies have shown a distinct recurrence and dissemination pattern to the CNS, skin, and testis.^3^, ^10^, ^11^

In the present study, we estimated the distribution and incidence of SNBCL subtypes and their characteristics, highlighting prognostic factors for the most common SNBCL – primary sinonasal DLBCL (primary sinonasal diffuse large B‐cell lymphoma (PSDLBCL)) – using survival analyses and regression models. We also investigated the treatment failure pattern of DLBCL, looking for a possible connection to specific extranodal sites.

## 2 METHODS

### 2.1 Study design

This nationwide cohort study included all adult patients (18 years or above) in Denmark (population approximately 5,800,000; 4,700,000 adults) with a registered diagnosis of B‐cell lymphoma in the nasal cavity or frontal, maxillary, ethmoid, and sphenoid sinuses. Patients were identified in the Danish Registry of Pathology from 1980 through 2018 using Systemized Nomenclature of Medicine codes. National archived and electronic healthcare records provided patient data. The Danish National Lymphoma Registry (LYFO) and the Cause of Death Register were used for missing data.^12^, ^13^ Formalin‐fixed, paraffin‐embedded specimens were collected, and reviewed by a trained hematopathologist Erik Clasen‐Linde using the latest WHO‐classification from 2017. The immunophenotype and cytogenetics of DLBCL, high‐grade B‐cell lymphoma (HGBCL) with or without double‐hit, or Burkitt lymphoma (BL) were validated using CD3, CD20, BCL2, BCL6, and cMYC with the addition of Epstein‐Barr virus‐encoded RNA in cases of BL, and all were analyzed for rearrangement of *MYC*. If *MYC*‐rearrangement was positive, samples were further analyzed for rearrangement of *BCL2* and *BCL6* (for further details on the validation of all lymphoma subtypes see Appendix S1).^14^ All samples of plasmacytoma were collected and validated, to ensure that all lymphomas were found. The majority of patients were presumed Caucasian due to the homogeneous composition of the Danish population.^15^

### 2.2 Clinical data

We collected data on sex, age at diagnosis, symptoms, duration of symptoms, elevated serum lactate dehydrogenase (LDH), location of relapse and dissemination, treatment and response,^16^ cause of death, and systemic involvement according to the Cotswolds‐modified Ann Arbor staging classification and the American Joint Committee on Cancer (American Joint Committee on Cancer) Lugano classification (Supplementary Table S1). Furthermore, the WHO/ECOG Performance Status was either extracted directly from medical record or estimated from the description of the patient, and the age‐adjusted International Prognostic Index was calculated. Stage, extension, and laterality were determined using clinical information, radiological reports, or available diagnostic imaging. *Primary SNBCL* was defined as a biopsy‐verified lymphoma with or without the involvement of regional lymph nodes (cervical or retropharyngeal) and no prior lymphoma. If a patient had lymphoma prior to involvement of the NPS or had disseminated lymphoma apart from the lesion in the NPS and associated regional lymph nodes, the patient would be classified as having a *secondary SNBCL*.

### 2.3 Statistics

Overall survival (OS) was defined as the period from diagnosis until death, whereas progression‐free survival (PFS) was until relapse, progression of refractory disease, or death, whichever occurred first. Overall survival, PFS, and cause‐specific absolute risk (AR) were calculated using the Kaplan‐Meier and Aalen‐Johansen estimators, with death from other causes considered a competing risk. The Gray's or logrank test were used to compare curves. We used the non‐parametric additive excess hazards model^17^ to compare mortality rates between the cohort and the general population matched by age, calendar year, and sex relative survival ratio (RSR). Life tables were downloaded from Statistics Denmark.^18^ The proportional excess hazards model was used to compare RSR curves, and hazard ratios (HRs) were calculated using the Cox proportional hazards model (“survival” and “timereg” packages, version 4.0.5, *R* core team).^19^, ^20^ Proportional hazards were tested using cumulative martingale residuals. If a variable was non‐proportional, the introduction of a cut‐off was attempted (“survival” package, “timeSplitter”),^19^ permitting a time‐varying effect. To ensure balance in follow‐up time, we based our regression model on a maximum of 10 years or until the last day of follow‐up: 31 December 2019. For survival analysis purposes, patients were categorized as having received chemotherapy if they underwent three or more cycles of therapy.

## 3 RESULTS

### 3.1 Inclusion and exclusion

Of the initial 295 registered patients with SNBCL and 39 patients with extraosseous plasmacytoma in the Danish national pathology registry, 205 patients with B‐cell lymphoma and 22 with plasmacytomas were included. None of the plasmacytomas were misdiagnosed. The number of patients with B‐cell lymphoma included per year and the reasons for exclusion are described in Supplementary Figure S1.

### 3.2 Lymphoma subtype classification and incidence

**FIGURE 1:**
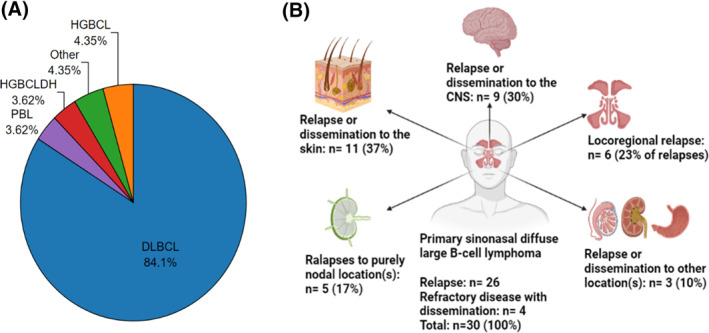
Subtype distribution of primary sinonasal lymphomas and recurrence/dissemination pattern for primary sinonasal diffuse large B‐cell lymphoma (PSDLBCL). (A) A clear majority of the primary lymphomas are of the subtype diffuse large B‐cell lymphomas (DLBCL). HGBCL, high‐grade B‐cell lymphoma (NOS); HGBCL (double hit) (HGBCLDH), high‐grade B‐cell lymphoma, HGBCL (double hit); PBL, plasmablastic lymphoma. “Other” is patients with follicular, lymphoplasmacytic, or Burkitt lymphoma (BL). (B) Diagram of sites of recurrence and sites of dissemination in the case of refractory primary sinonasal DLBCL

Ten histologically distinct B‐cell lymphoma subtypes were identified, with DLBCL being the predominant subtype (*n* = 163; 80%). Aside from DLBCL, six other subtypes presented as primary lymphomas (Figure 1). Frequency, clinical characteristics, and prognosis for all subtypes are described in detail in Table 1 and Supplementary Table S3.

**TABLE 1:** Clinical and demographic characteristics and treatment response

	All *n* (%)	DLBCL *n* (%)	HGBCL *n* (%)	HGBCLDH *n* (%)	MCL *n* (%)	PBL *n* (%)	Other *n* (%)
Total	205	163	8	8	5	5	16
% Of all lymphomas	100.0	79.6	3.9	3.9	2.4	2.4	7.8
Male	126 (62)	98 (60)	3 (38)	6 (75)	4 (80)	5 (100)	10 (63)
Age at diagnosis, years							
Median [range], years	72.0 [18.6–100.2]	72.8 [18.6–100.7]	73.4 [52.4–89.6]	69.6 [54.2–85.4]	64.3 [49.2–77.9]	66.1 [30. 6–78.1]	69.2 [29.2–86.6]
Disease group							
Primary lymphoma without lymph node involvement	116 (56)	101 (62)	2 (25)	5 (62)	‐	4 (80)	4 (25)
Primary lymphoma with lymph node involvement	22 (11)	15 (9)	4 (50)	‐	‐	1 (20)	2 (12)
Secondary lymphoma	60 (30)	40 (25)	2 (25)	3 (38)	5 (100)	‐	10 (63)
Unknown	7 (3)	7 (4)	‐	‐	‐	‐	‐
Sinonasal region as presenting site							
Yes	150 (72)	118 (72)	7 (88)	7 (88)	3 (60)	4 (80)	11 (69)
No	24 (12)	15 (9)	1 (12)	1 (12)	2 (40)	1 (20)	4 (25)
Unknown	31 (16)	30 (19)	‐	‐	‐	‐	1 (6)
Performance status							
0	122 (59)	93 (57)	4 (50)	6 (75)	4 (80)	5 (100)	10 (63)
1	51 (25)	43 (26)	3 (38)	1 (12)	‐	‐	4 (25)
2	15 (7)	14 (9)	‐	‐	1 (20)	‐	‐
3	4 (2)	3 (2)	‐	‐	‐	‐	1 (6)
4	2 (1)	‐	1 (12)	1 (12)	‐	‐	‐
Unknown	11 (6)	10 (6)	‐	‐	‐	‐	‐
Elevated LDH							
No	140 (68)	109 (66)	6 (76)	8 (100)	3 (60)	3 (60)	11 (69)
Yes	34 (16)	28 (17)	1 (12)	‐	1 (20)	1 (20)	3 (19)
Unknown	31 (16)	26 (17)	1 (12)	‐	1 (20)	1 (20)	2 (12)
Site							
Nasal cavity	76 (37)	58 (35)	3 (38)	5 (62)	2 (40)	2 (40)	6 (38)
Maxillary sinus	70 (33)	52 (32)	5 (62)	2 (25)	2 (40)	3 (60)	6 (38)
Ethmoid sinus	13 (7)	12 (8)	‐	‐	‐	‐	1 (6)
Sphenoid sinus	4 (2)	4 (2)	‐	‐	‐	‐	‐
Frontal sinus	3 (2)	2 (1)	‐	1 (13)	‐	‐	‐
Multiple	25 (12)	21 (13)	‐	‐	1 (20)	‐	3 (18)
Sinus unspecified	14 (7)	14 (9)	‐	‐	‐	‐	‐
Laterality							
Unilateral	174 (84)	138 (85)	8 (100)	8 (100)	4 (80)	4 (80)	13 (82)
Bilateral	20 (10)	15 (9)	‐	‐	1 (20)	1 (20)	2 (12)
Unknown	11 (6)	10 (6)	‐	‐	‐	‐	1 (6)
B symptoms							
No	151 (73)	121 (74)	7 (88)	7 (88)	2 (40)	2 (40)	12 (75)
Yes	30 (15)	22 (14)	1 (12)	1 (12)	2 (40)	2 (40)	4 (25)
Unknown	24 (12)	20 (12)	‐	‐	1 (20)	1 (20)	‐
Ann Arbor stage							
IE	123 (59)	107 (65)	2 (25)	6 (75)	‐	4 (80)	4 (25)
IIE	31 (15)	24 (15)	4 (50)	‐	‐	1 (20)	2 (12)
III	8 (4)	5 (3)	1 (12)	‐	‐	‐	2 (12)
IV	36 (18)	20 (12)	1 (12)	2 (25)	5 (100)	‐	8 (50)
Unknown	7 (4)	7 (5)	‐	‐	‐	‐	‐
AJCC							
IE	123 (59)	107 (65)	2 (25)	6 (75)	‐	4 (80)	4 (25)
IIE	24 (11)	17 (10)	4 (50)	‐	‐	1 (20)	2 (12)
IV	51 (25)	32 (20)	2 (25)	2 (25)	5 (100)	‐	10 (63)
Unknown	7 (5)	7 (5)	‐	‐	‐	‐	
Age‐adjusted IPI							
0	105 (51)	88 (54)	5 (63)	6 (75)	‐	3 (60)	3 (19)
1	50 (24)	34 (21)	1 (12)	1 (12)	3 (60)	1 (20)	10 (63)
2	16 (8)	14 (9)	1 (12)	1 (12)	1 (20)	‐	‐
3	3 (2)	1 (<1)	0	0	‐	‐	1 (6)
Unknown	31 (15)	26 (16)	1 (12)	0	1 (20)	1 (20)	2 (12)
>1 extranodal site							
Yes	28 (13)	18 (11)	1 (13)	2 (25)	3 (60)	‐	8 (50)
Relapse/progression of lymphoma							
No relapse or progression	107 (52)	83 (51)	6 (75)	6 (75)	1 (20)	4 (80)	7 (44)
Relapse from primary lymphoma	29 (14)	26 (16)	1 (12)	‐	‐	1 (20)	1 (6)
Relapse from secondary lymphoma	15 (9)	10 (7)	‐	‐	3 (60)	‐	2 (12)
Progression of refractory lymphoma	24 (10)	16 (9)	1 (12)	1 (12)	1 (20)	‐	5 (32)
Unknown	30 (15)	28 (17)	0	1 (12)	‐	‐	1 (6)
Response							
Complete response	139 (68)	109 (66)	6 (75)	6 (75)	3 (60)	5 (100)	10 (64)
Partial response	13 (7)	10 (7)	‐	1 (12)	‐	‐	2 (12)
Stable disease	25 (12)	20 (12)	1 (12)	0	1 (20)	‐	3 (18)
Died before evaluation	17 (8)	13 (8)	1 (12)	1 (12)	1 (20)	‐	1 (6)
Unknown	11 (5)	11 (7)	‐	‐	‐	‐	‐
Status at last follow‐up							
Alive with complete remission	64 (32)	50 (31)	4 (50)	4 (50)	1 (20)	3 (60)	2 (11)
Alive with disease	14 (6)	2 (1)	3 (38)	2 (25)	1 (20)	1 (20)	5 (32)
Dead from lymphoma	82 (41)	71 (44)	1 (12)	2 (25)	3 (60)	1 (20)	4 (25)
Dead from other cause	45 (21)	40 (24)	‐	‐	‐	‐	5 (32)
Median time to death from lymphoma	0.96 [0.01–10.8]	0.96 [0.01–10.8]	0.57 [0.5–6.7]	1.57 [0.3–2.8]	0.5 [0.2–6.8]	3.99 [NA]	4.8 [0.3–25.5]

Abbreviations: AJCC, American Joint Committee on Cancer; DLBCL, diffuse large B‐cell lymphoma; HGBCL, high‐grade B‐cell lymphoma (NOS); HGBCL (double hit) (HGBCLDH), HGBCL (double hit); IPI, International Prognostic Index; MCL, Mantle cell lymphoma; PBL, plasmablastic lymphoma. “Other” comprises the following subtypes: follicular‐, Burkitt‐, extranodal marginal zone B‐cell‐, LGBCL‐, and lymphoplasmacytic‐lymphoma (Supplementary Table 3). Percentages may only add up to 99% due to the rounding of numbers.

Due to the completeness of the data from 2009 and onward (Supplementary Figure S1), we were able to calculate the national incidence of SNBCL: 8/year/5,800,000 people (0.14/100,000 person‐years). Based on the average of new cases of non‐Hodgkin lymphoma (NHL) from 2014 to 2018 found in LYFO, the national incidence of NHL was 22.9/100,000 person‐years, which means that SNBCL constituted 0.61% of all NHLs in Denmark.^21^

### 3.3 Patient characteristics

#### 3.3.1 All subtypes

The median age at diagnosis was 72; 126 (62%) patients were male. Performance Status scores were evenly distributed between subtypes, with a majority having zero or one (*n* = 153; 84%). In 34 cases (16%), serum LDH at diagnosis was above the upper limit, according to the reference interval of the time. Table 1 contains detailed information about the five most prevalent subtypes; Supplementary Table S3 describes all other lymphomas in detail.

#### 3.3.2 All diffuse large B‐cell lymphoma

In the 163 patients with DLBCL, lesions were primarily unilateral to the nasal cavity (*n* = 58; 38%) or maxillary sinus (*n* = 52; 32%). Upon diagnosis, widespread extension to other adjacent anatomical structures was common. Apart from other sinonasal compartments, the foremost affected structure was the orbit. Extension from a lesion in the maxillary, ethmoid, or sphenoid sinuses to the orbit was present in 38%, 38%, and 75% of cases, respectively (Supplementary Table S2).

Of the 116 patients with PSDLBCL, 60% were male and the median age was 74. Primary sinonasal diffuse large B‐cell lymphoma constituted 84% of all primary SNBCLs. Patients with PSDLBCL resembled the rest of the cohort, with 106 (91%) patients having a performance status of zero or 1. Fifteen patients presented with contiguous spread to cervical lymph nodes (12%).

Disseminated or prior lymphoma (secondary sinonasal DLBCL) was present in 28 (17%) and 12 (8%) cases. In 9 of 12 relapsed cases, earlier lymphomas were purely extranodal (two breast, two skin, two testis, three CNS, and one bone marrow). Relapses to the NPS were purely extranodal in 92% of cases (eight with NPS alone and two with NPS and involvement of either skin or breast). Disseminated lymphomas were solely extranodal in eight cases (29%; three skin, one CNS, one kidney, one lung, and one bone marrow), purely nodal apart from sinonasal involvement in 11 cases (39%; six with IIE and five with III), and in nine cases (32%) the sinonasal lymphoma was accompanied by both extranodal (testis, skin, ovaries, stomach, and colon) and nodal lesions, often in relation to extranodal sites.

#### 3.3.3 Other lymphomas

Other lymphomas constituted 42 patients (21%) in the cohort. Characteristic for patients with rarer subtypes was more widespread disease at the time of diagnosis compared with DLBCL and HGBCL, regardless of double‐hit status. Peripheral blood involvement was found in one patient with mantle cell lymphoma and one with low‐grade B‐cell (LGBCL) lymphoma (not otherwise specified; Table 1 and Supplementary Table S3).

### 3.4 Symptoms

#### 3.4.1 All subtypes

With 80 patients experiencing nasal congestion (39%), this was the most dominant symptom across all subtypes. Other common symptoms were facial swelling (24%) and pain (23%). Although subtypes did not show substantial differences in symptoms, epistaxis was more prevalent in aggressive lymphomas (13%–25%) and lymphoplasmacytic lymphomas (50%). Sinonasal symptoms were the first presentation of the disease in 72% vs. 12% of all lymphoma cases. The median time from symptom onset until diagnosis was 2 months (Table 2).

**TABLE 2:** Sinonasal symptoms according to lymphoma subtype

	Predominant subtypes						Other subtypes				
	All (%)	DLBCL	HGBCL	HGBCLDH	MCL	PBL	FL	BL	EMZL	LPL	LGBCL
Total (100%)	206	163	8	8	5	5	4	4	3	3	2
Symptoms (% of patients)											
First presenting symptom in sinonasal region	150 (73)	118 (72)	7 (88)	7 (88)	3 (60)	4 (80)	3 (75)	‐	2 (66)	2 (50)	1 (50)
Epistaxis	27 (13)	23 (14)	1 (13)	‐	‐	‐	‐	1 (25)	‐	2 (50)	‐
Congestion	80 (39)	64 (39)	3 (38)	5 (63)	1 (20)	3 (60)	‐	1 (25)	1 (33)	1 (25)	1 (50)
Pain	48 (23)	38 (23)	1 (13)	1 (13)	1 (20)	2 (40)	1 (25)	3 (75)	‐	1 (25)	‐
Facial swelling	50 (24)	34 (21)	4 (50)	3 (38)	3 (60)	2 (40)	2 (50)	‐	‐	2 (50)	‐
Epiphora	16 (8)	15 (9)	‐	‐	‐	‐	‐	‐	‐	1 (25)	‐
Change in facial sensory function	20 (10)	16 (10)	2 (25)	‐	‐	‐	2 (50)	‐	‐	‐	‐
Exophthalmos	23 (11)	16 (10)	1 (13)	1 (13)	‐	1 (20)	1 (25)	1 (25)	‐	2 (50)	‐
Diplopia	30 (15)	23 (14)	1 (13)	1 (13)	‐	1 (20)	1 (25)	2 (50)	‐	1 (25)	‐
Loose teeth	4 (2)	3 (2)	1 (13)	‐	‐	‐	‐	‐	‐	‐	‐
Nasal discharge	10 (5)	9 (6)	‐	‐	1 (20)	‐	‐	‐	‐	‐	‐
Vision impairment	5 (3)	5 (3)	‐	‐	‐	‐	‐	‐	‐	‐	‐
Other	11 (5)	6 (4)	‐	1 (13)	2 (40)	‐	1 (25)	‐	1 (33)	‐	‐
Not stated	16 (8)	13 (8)	‐	1 (13)	1 (20)	‐	‐	‐	‐	1 (25)	
Median symptom duration before diagnosis, [range] (mos.)	2 [0.3; 24]	2.5 [0.3; 12]	1 [1; 2.5]	2 [0.5; 3]	5 [2; 18]	2 [1; 8]	7 [3; 9]	1 [0.5; 1]	24 [NA]^a^	1.5 [1; 2]	12 [NA]^a^

*Note*: Sum of percentages >100% due to multiple symptoms per patient. Other symptoms: crusts, impaired hearing, dyspnea, toothache, anosmia/hyposmia. DLBCL, diffuse large B‐cell lymphoma; HGBCL, high‐grade B‐cell lymphoma (NOS); HGBCLDH, high‐grade B‐cell lymphoma (double hit); MCL, Mantle cell lymphoma; PBL, plasmablastic lymphoma; FL, follicular lymphoma; BL, Burkitt lymphoma, EMZL extranodal marginal zone B‐cell lymphoma; LGBL, low‐grade B‐cell lymphoma; LPL, lymphoplasmacytic lymphoma.

^a^ Duration only available for one patient.

### 3.5 Treatment and outcome

#### 3.5.1 All subtypes

Out of 205 patients 147 received CHOP (cyclophosphamide, doxorubicin, vincristine, and prednisone) or CHOP‐like regimens as initial treatment. Other chemotherapy regimens were given in 12 cases. Across all subtypes, consolidative radiotherapy was utilized for low‐stage disease (EI and EII; *n* = 68), with patients receiving 30–40 Gray over 15 or 20 fractions.

CNS prophylaxis was given in 64 cases spread across all stages, consisting of either high‐dose methotrexate (73%), cytarabine (8%), a combination of methotrexate and cytarabine (13%), or triple CNS prophylaxis (methotrexate, cytarabine, and prednisolone; 6%). Overall, for the largest subtype, DLBCL, 123 (75%) patients out of the 163 cases received CHOP or CHOP‐like regimens, and 8 (5%) received other regimens. Of those treated with chemotherapy or immunochemotherapy, 68 (41%) received additional radiotherapy and 51 (31%) CNS‐prophylaxis. Detailed information on initial treatment specifically for sinonasal DLBCL is provided in Supplementary Table S4.

#### 3.5.2 Primary sinonasal diffuse large B‐cell lymphoma (PSDLBCL)

Chemotherapy was administered in 93 of 116 cases (44 received three to five cycles and 49 > 5 cycles); 53 received additional immunotherapy. Overall, a complete response was observed in 83% of cases; 5% and 4% had a partial or no response. Six died before response evaluation. Recurrence and dissemination/locoregional progression was observed in 26 (22%) and 7 (6%) patients, respectively. Recurrence was extranodal in 20 cases (77%), purely nodal in five (19%), and both in one case (4%). Extranodal sites included six NP, nine skin on arms and legs, seven CNS, one stomach, and one affecting one testis. Progression of refractory disease was at the following sites: three had locoregional progression; one disseminated to the skin and kidney, one to the skin and testis, and two to the CNS (Figure 1). The median time until relapse or until locoregional progression or dissemination was 3.4 years (0.71–15.35) and 0.55 years (0.24–1.26), respectively.

**FIGURE 2:**
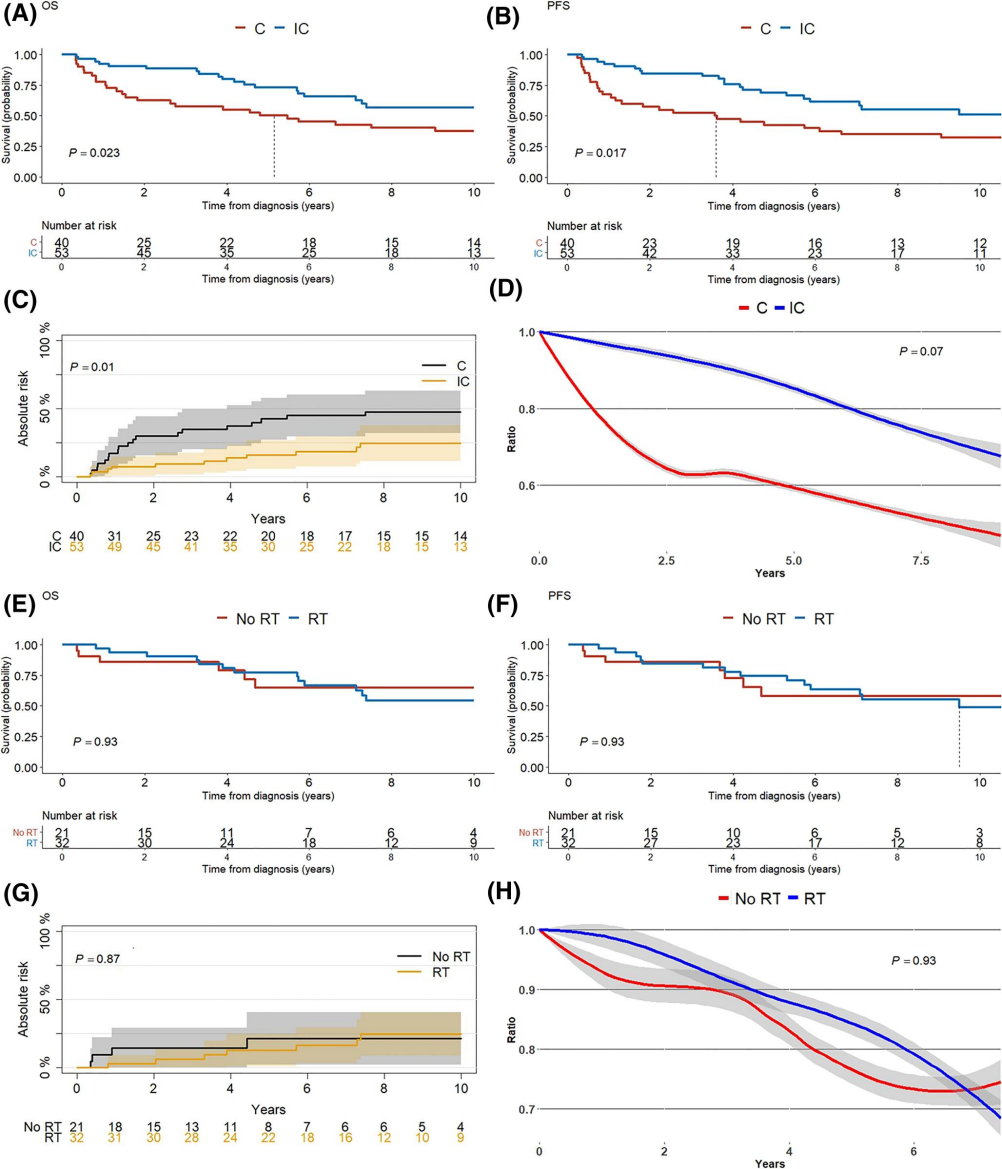
Patients with primary diffuse large B‐cell lymphoma (DLBCL) treated with chemotherapy and immunochemotherapy, stratified by immunotherapy and radiotherapy, respectively (A–D) Patients receiving chemotherapy, stratified by immunotherapy. (A) overall survival (OS). (B) progression‐free survival (PFS). (C) absolute risk (AR) of dying from primary sinonasal diffuse large B‐cell lymphoma (PSDLBCL) (*p* = 0.01). (D) Relative survival ratio, that is, the ratio of mortality compared with the background population adjusted for age, calendar year and sex (*p* = 0.07). (E–H) Patients receiving immunochemotherapy, stratified by consolidative radiotherapy. (E) OS (F) PFS. (G) AR of dying from PSDLBCL. (H) Relative survival ratio (*p* = 0.95). Dotted vertical line indicates median survival. C, chemotherapy (chop or chop‐like); IC, immunochemotherapy; RT, radiotherapy

With a median follow‐up time of 5.7 years, five‐year survival rates for all PSDLBCLs were: OS, 56%; PFS, 50%, AR 33%; and RSR 0.67. Five‐year survival rates stratified by chemotherapy vs. immunochemotherapy were: OS 50% vs. 73%, *p* = 0.023; PFS 36% vs. 67%, *p* = 0.023; and AR 42% vs. 16%, *p* = 0.01. Although not statistically significant, the five‐year RSR showed an effect of immunotherapy on survival (0.85 vs. 0.60, *p* = 0.07; see Figure 2). Consolidative radiotherapy in addition to immunochemotherapy (32 received; 21 did not) did not show any additional benefit: OS, *p* = 0.93; PFS, *p* = 0.93; AR, *p* = 0.87; and RSR, *p* = 0.51 (Figure 2). Age above 60 (HR 4.98, *p < *0.01) as well as performance status 1 (HR 1.27, *p* < 0.001) were statistically significant negative prognostic factors, while lymph node involvement trended toward significance (HR 2.18, *p* = 0.09). The addition of immunotherapy reduced the hazard of death by 72% (*p* < 0.001). Consolidative radiotherapy violated the proportionality assumption; cumulative martingale residuals showed a reasonable cut‐off at 1 year (Supplementary Figure S2). Hazard ratio from zero to 1 year and after 1 year were, *p* < 0.01 and 1.76, *p* = 0.17 (Table 3).

**TABLE 3:** Univariate and multivariate regression analysis on overall survival (OS)

	Univariate HR (95% Cl)	*p*	PH	Multivariate HR (95% Cl)	*p*	PH
**Patient characteristics**						
***Age***						
<60 years	REF			REF		
>60 years	3.63 (1.12–1.70)	*<0.01*	0.31	4.98 (1.48–16.80)	*<0.01*	0.21
***Performance status***						
0	REF			REF		
1	2.97 (1.58–5.58)	*<0.001*	0.41	3.57 (1.81–7.03)	*<0.001*	0.59
2–3	5.84 (1.96–17.44)	*0.01*	0.15	2.43 (0.71–8.24)	0.15	0.17
***Sex***						
Male						
Female	0.56 (0.30–1.07)	0.08	0.41	0.55 (0.28–1.10)	0.09	0.27
**Disease burden**						
**Regional lymph node involvement**						
No	REF			REF		
Yes	1.99 (0.96–4.15)	0.07	0.51	2.18 (0.87–5.12)	0.09	0.37
***Elevated LDH***						
No	REF			‐		
Yes	0.95 (0.37–2.45)	0.92	0.32	‐		
***Transformation***						
No	REF			‐		
Yes	1.07 (0.38–2.98)	0.90	0.19	‐		
Treatment						
Rituximab – No	REF			REF		
Rituximab ‐ Yes	0.51 (0.28–0.92)	*0.03*	0.20	0.29 (0.14–0.57)	*<0.001*	0.24
Radiotherapy ‐ No	REF			REF		
Radiotherapy ‐ Yes	0.9 (0.49–1.66)	0.12	0.01^a^			
Radiotherapy – Yes <1 year	‐			0.21 (0.05–0.83)	*0.03*	
Radiotherapy – Yes >1 year	‐			1.76 (0.79–3.92)	0.16^b^	

*Note*: LDH, lactate dehydrogenase; REF, reference; PH: *p*‐value testing for proportional hazard.

^a^ Covariate shows non‐proportionality of hazard ratio (HR).

^b^ Time *x* radiotherapy *p* = 0.001, indicating a continued interaction with time.

#### 3.5.3 Secondary Diffuse large B‐cell lymphoma

Chemotherapy was administered in 29 cases; 16 received additional treatment with Rituximab. Treatment response was complete in 21 patients (50%) and partial in 5 (13%); 5 patients (12%) showed no effect, and two (5%) died before evaluation.

Recurrences were equally distributed between patients with or without prior lymphoma. Six recurrences were extranodal (one NPS, three skin, two CNS, and one pancreas), and three were nodal (cervical or mediastinal lymph nodes); one was both (skin and multiple lymph nodes). Eight cases had progression: one locoregionally, one with purely nodal, and six with extranodal dissemination (three skin, two CNS, two breast, and one bone marrow). The median time until relapse or locoregional progression/dissemination was 3.1 years (0.59–11.49) and 0.54 years (0.08–0.94), respectively.

## 4 DISCUSSION

To our knowledge, this is the first nationwide population‐based study to establish valid clinical data on the incidence, anatomical distribution, presentation, treatment failure pattern, and survival prognosis on a grand scale relative to the rarity of SNBCLs. We found that DLBCL was the dominant subtype by a sizable majority and that the incidence of SNBCL was 0.14/100,000 person‐years, which translates to 0.61% of all NHLs per year. This compares well with rates found in a recent Finnish single‐center study on validated sinonasal tract lymphomas, though rates were based on pooled nodal nasopharyngeal lymphomas, known to have higher rates of “small B‐cell lymphomas” (e.g., Mantle cell‐ and follicular lymphoma), and extranodal sinonasal lymphomas.^22^, ^23^

High‐grade B‐cell lymphomas with *MYC* and *BCL2* and/or *BCL6* rearrangements were rare in the NPS, which is clinically relevant because these lymphomas have a poor prognosis.^24^ The distribution of subtypes found in the NPS stands in stark contrast to the adjacent orbital region, where extranodal marginal zone B‐cell lymphoma represents approximately 56% of all lymphomas,^25^ emphasizing the non‐randomness of the localization of extranodal lymphomas.

In most cases, sinonasal symptoms were the first presentation, even for patients with late‐stage disease. If a lymphoid lesion in the NPS is found, it is likely a primary SNBCL, as two‐thirds of patients had localized disease at the time of diagnosis. If regional lymph nodes were involved, it was through contiguous spread to cervical lymph nodes; none had involvement of retropharyngeal lymph nodes. Thus, persistent cervical lymphadenopathy should be followed by an examination of the NPS, keeping sinonasal symptoms in mind when taking the patient's history, particularly if the patient exhibits typical symptoms of rhinosinusitis.

We were able to explore the differences in survival between specific treatment groups due to the significant time span of our data, covering both the pre‐ and post‐rituximab era. Our data showed how contemporary treatment improved the prognosis of PSDLBCL; this was further validated both in the RSR and AR analysis. The regression analysis revealed that immunotherapy reduced the hazard of death by 78% after adjusting for multiple covariates (Table 3), illustrating the change in survival in the post‐rituximab era. Overall, survival analyses did not show any effect of consolidative radiotherapy, although we found a significant reduction in the hazard of death in the first year after diagnosis. This may be due to intervention bias, as patients who do not experience a high level of toxicity from chemotherapy are more inclined to also undergo consolidative radiotherapy. In the literature, both in cohort studies on lymphomas of related sites (e.g., early‐stage extranodal DLBCL of the head and neck and sinonasal tract DLBCL stage IE and IIE) and in randomized controlled trials on localized lymphoma, consolidative radiotherapy seems to have a limited effect.^26^, ^27^, ^28^, ^29^ In a meta‐analysis from 2021 on the use of consolidative radiotherapy, the authors found randomized controlled trials to be too heterogeneous in terms of stage, subtype, and nodal/extranodal involvement to draw any solid conclusions and, when only analyzing studies of early‐stage disease, they found radiotherapy to have no effect on the PFS.^26^ Furthermore, because PSDLBCL is a disease of the elderly and curable with pharmacological treatment, the efficacy of radiotherapy must be weighed against its toxicity and the rate of locoregional recurrence. Although drawing conclusions on treatment based upon retrospective data is not recommended, this may be the closest we come to hard evidence regarding the use of consolidative radiotherapy for PSDLBCL, as the rarity of the disease makes prospective studies or randomized controlled trials difficult.

In the regression analysis, apart from the more apparent predictors of death (e.g., age over 60 and performance status), involvement of regional lymph nodes seemed to increase the hazard by a factor of 2.18 (95% CI 0.87–5.12). Though statistically non‐significant, the data indicated that cervical lymph node involvement trends toward posing a risk for patients with PSDLBCL. This means that our results support cervical lymph node diagnostics in cases of suspicious lymphadenopathy in connection with a lymphoma of the NPS to ascertain the extension and prognosis of the disease. However, the more likely metastasis from a squamous cell carcinoma of the head and neck should be ruled out by extirpation of lymph nodes if a fine needle biopsy is non‐conclusive.^30^

Although only one recent study on the treatment failure pattern of primary DLBCL of the nasal cavity has been conducted, our study found the same extranodal pattern for both primary and secondary nasal and paranasal DLBCL: skin, CNS, locoregional involvement, and testis. Furthermore, we included lymphomas of the breast to the list, though these were only featured in secondary disease. This pattern may be due to specific mutational subtypes such as the MCD (based on the co‐occurrence of *MYD88* ^L265P^ and *CD79 B* mutations), as seen in previous studies of primary CNS, testicular, breast, and cutaneous DLBCL.^10^ As this is outside the scope of this study, future studies will have to focus on mechanisms, which may be driving factors in the specific homing/survival of malignant B‐cells in the histologically distinct sinonasal mucosa.

Like all registry‐based publications, the present study is subject to inherent limitations. Although only 25% of cases are from before the year 2000, older cases may have been staged with a lower degree of certainty. Furthermore, we did not have enough patients to stratify by cycles of chemotherapy, which may have influenced the efficacy of radiotherapy. Moreover, in the regression analysis, we were not able to consider the differences in radiotherapy over the years.

In conclusion, the incidence of SNBCL is 0.14/100,000 person‐years, 0.61% of all NHLs. Diffuse large B‐cell lymphoma is the most prevalent SNBCL, mainly presenting as primary lymphoma, although nine other subtypes were represented. Treatment with immunotherapy has improved the prognosis for PSDLBCL, while consolidative radiotherapy did not seem beneficial. Treatment failure patterns showed specific sites (i.e., skin, breast, CNS, and testis) where recurrence or dissemination of DLBCL occurred, suggesting a possible common pathogenesis.

## CONFLICT OF INTEREST

The authors declare that the research was conducted in the absence of any commercial or financial relationships that could be construed as potential conflicts of interest.

## ETHICS STATEMENT

Our study was approved by the Regional Committee on Health Research Ethics (journal no. H‐16023080) and the Danish Data Protection Agency (journal no. P‐2020‐588).

### PEER REVIEW

The peer review history for this article is available at https://publons.com/publon/10.1002/hon.2968.

## Supporting information

Supplementary Material 1

Figure S1

Figure S2

Table S1

Table S2

Table S3

Table S4

## Data Availability

The data that support the findings of this study are available on request from the corresponding author. The data are not publicly available due to privacy or ethical restrictions.
