# Supplementary material for: Sinonasal B‐cell lymphomas: A nationwide cohort study, with an emphasis on the prognosis and the recurrence pattern of primary diffuse large B‐cell lymphoma
Source: Hematol Oncol. 2022 Feb 6;40(2):160–71. doi: 10.1002/hon.2968 (PMC9303446; doi:10.1002/hon.2968)
Supplement: Supplementary file 1 — Supplementary Material 1 [file HON-40-160-s003.docx]

Appendix S1. Validation process.

**Diffuse large b-cell lymphoma (DLBCL) and high-grade B-cell lymphoma with MYC and BCL2 and/or BCL6 rearrangement (HGBCLDH)**

*Immunohistochemistry*: CD3, CD20, BLC2, BLC6, cMYC.

All lymphomas with DLBCL morphology were subjected to further cytogenetic analysis. Whether the lymphoma was classified as DLBCL or HGBCLDH was determined by cytogenetic analysis with fluorescence in situ hybridization (FISH), where rearrangement in *MYC* and *BCL2* and/or *BCL6* was mandatory for a diagnosis of HGBCLDH; otherwise, the lymphoma was classified as DLBCL.

**High-grade B-cell lymphoma (HGBCL)**

If the morphology had features of Burkitt lymphoma (BL) and DLBCL or a blastoid appearance, the lymphoma was analyzed with FISH to determine whether it was an HGBCLDH or BL. Otherwise, the lymphoma was classified as HGBCL.

**Extranodal marginal zone B-cell lymphoma (EMZL), plasmablastic lymphoma (PBL) and lymphoplasmacytic lymphoma (LPL)**

*Immunohistochemistry*: CD3, CD5, CD10, CD20, CD23, BCL2, BCL6, CyclinD1.

All extraosseous plasmacytomas were stained for CD138 and Kappa/Lambda in situ hybridization or immunohistochemistry. If the morphology did not portray extraosseous plasmacytoma, the microarrays were further tested for MUM1, CD3, and CD20 to determine whether the lymphoma was a PBL.

**Burkitt lymphoma (BL)**

Immunohistochemistry: CD3, CD10, CD20, BCL2, BCL6, cMYC, EBER.

All potential BLs were cytogenetically analyzed using FISH. The lymphoma would have to be positive for rearrangement in *MYC* and negative for rearrangement in *BCL2* and *BCL6*.

**Mantle cell lymphoma (MCL)**

*Immunohistochemistry*: CD 3, CD5, CD 20, CyclinD1, SOX11.

**Follicular lymphoma (FL)**

*Immunohistochemistry*: CD3, CD10, CD20, BCL2, BCL6.

**Low-grade B-cell lymphoma (LowGBCL)**

*Immunohistochemistry*: CD3, CD20, CD10, BLC2, BCL6, CyclinD1, SOX11.

This term was used in one instance when we were not able to attain a subtype and the morphology did not conform to that of an aggressive lymphoma.

**Fluorescence in situ hybridization (FISH)**

In addition to the routine diagnostic immunohistochemistry, fluorescence in situ hybridization (FISH) was performed using break-apart probes for *MYC*. Analysis for rearrangement for *BCL2* and *BCL6* was subsequently performed if *MYC* was positive. Rearrangement analysis was performed following the manufacturer’s protocol using the HYBrite platform (Abbott Molecular). After hybridization, nuclei were counterstained with DAPI II (ZytoVision), and one hundred nuclei were counted. Only nuclei whose entire nuclear membrane was visualized were scored. The positive cut-off value was 10%.
