# Supplementary material for: Sinonasal B‐cell lymphomas: A nationwide cohort study, with an emphasis on the prognosis and the recurrence pattern of primary diffuse large B‐cell lymphoma
Source: Hematol Oncol. 2022 Feb 6;40(2):160–71. doi: 10.1002/hon.2968 (PMC9303446; doi:10.1002/hon.2968)
Supplement: Supplementary file 2 — Figure S1 [file HON-40-160-s001.docx]

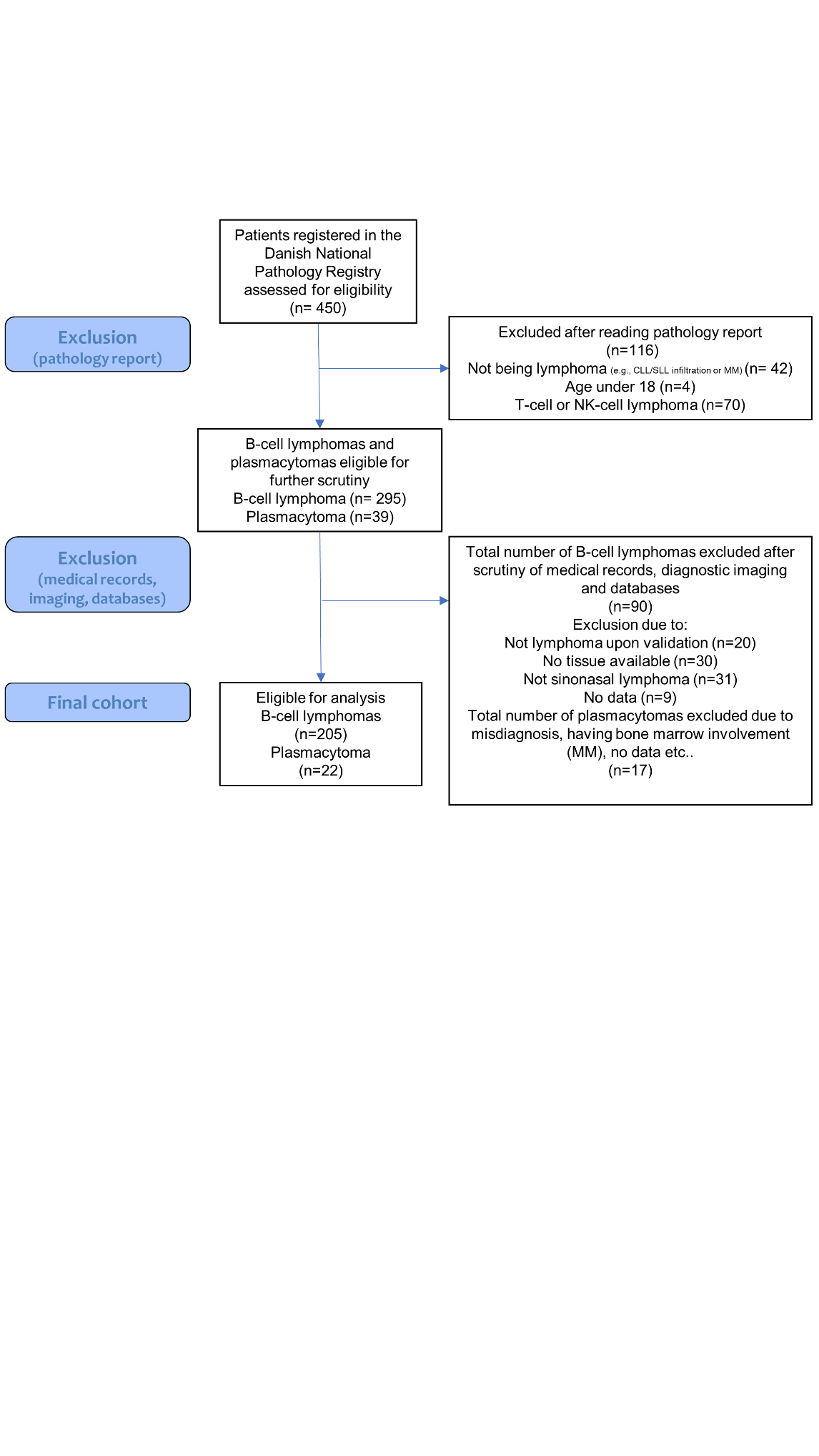


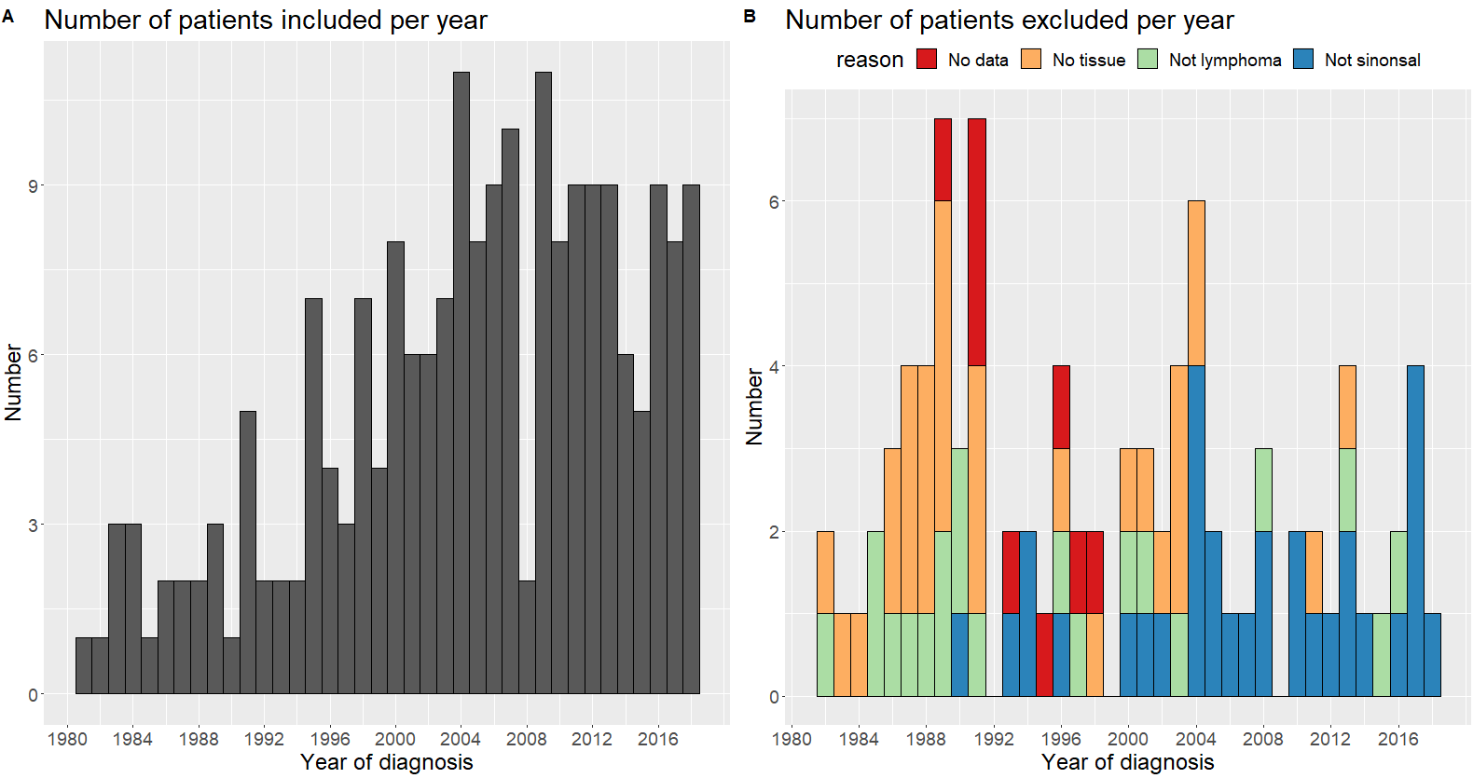


**Supplementary Figure S1. Inclusion and exclusion of patients.** We collected tissue and medical records/registry data from 295 adult patients. A total of 205 B-cell lymphomas and 22 extraosseous plasmacytomas were included. Extraosseous plasmacytomas are not included in the histogram. Above: flow diagram of inclusion process. Below: (A) All included B-cell lymphomas, by year. (B) All excluded B-cell lymphomas by year (color indicates reason for exclusion). The completeness of data from 2009 until 2019 enabled us to calculate the incidence of sinonasal lymphoma in Denmark (8/year/5.8M people, or 0.14/100,000 person-years). CLL, Chronic lymphatic leukemia; MM, multiple myeloma.
