# Supplementary material for: Sinonasal B‐cell lymphomas: A nationwide cohort study, with an emphasis on the prognosis and the recurrence pattern of primary diffuse large B‐cell lymphoma
Source: Hematol Oncol. 2022 Feb 6;40(2):160–71. doi: 10.1002/hon.2968 (PMC9303446; doi:10.1002/hon.2968)
Supplement: Supplementary file 3 — Figure S2 [file HON-40-160-s002.docx]

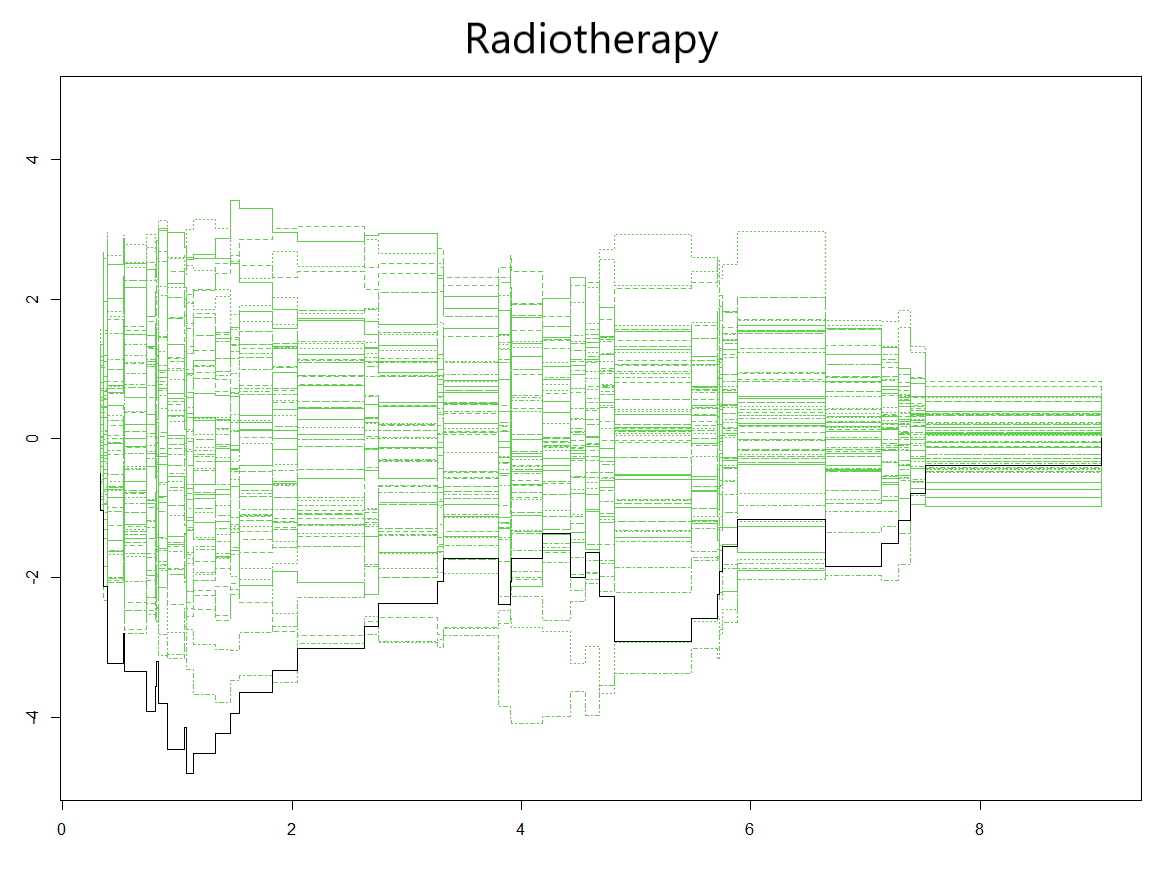


**Supplementary Figure S2. Proportionality and cumulative martingale residuals.** Black line: Cumulative martingale residuals for covariate. Green line: Fifty simulated cumulative residuals for covariate if it was proportional. Radiotherapy seems to have a more positive effect than expected for the first year. All other covariates were proportional.
