# Supplementary material for: Sinonasal B‐cell lymphomas: A nationwide cohort study, with an emphasis on the prognosis and the recurrence pattern of primary diffuse large B‐cell lymphoma
Source: Hematol Oncol. 2022 Feb 6;40(2):160–71. doi: 10.1002/hon.2968 (PMC9303446; doi:10.1002/hon.2968)
Supplement: Supplementary file 4 — Table S1 [file HON-40-160-s005.docx]

Supplementary Table S1. Staging of lymphoma.

| Description of Extent | AJCC Staging Stage | Ann Arbor Staging |
| --- | --- | --- |
| A single extralymphatic organ or site | IE | IE |
| A single extralymphatic organ and its regional lymph nodes | IIE | IIE |
| Localized involvement of one extralymphatic organ and involvement of non-regional lymph nodes on the same side of the diaphragm with or without regional lymph node involvement | IV | IIE |
| Involvement of lymph node regions on both sides of the diaphragm plus localized involvement of an extralymphatic organ or site | IV | III |
| Disseminated involvement of multiple extralymphatic sites with or without lymph node involvement | IV | IV |
| Isolated extralymphatic organ involvement with distant (non-regional) nodal involvement | IV | IV |
