# Supplementary material for: Sinonasal B‐cell lymphomas: A nationwide cohort study, with an emphasis on the prognosis and the recurrence pattern of primary diffuse large B‐cell lymphoma
Source: Hematol Oncol. 2022 Feb 6;40(2):160–71. doi: 10.1002/hon.2968 (PMC9303446; doi:10.1002/hon.2968)
Supplement: Supplementary file 5 — Table S2 [file HON-40-160-s004.docx]

Supplementary Table S2. Extension of sinonasal DLBCL to other adjacent anatomical regions, by primary site.

| Primary site (% of lymphomas originating from site) | Total by site of origin | Isolated to site | Orbit | Oral cavity | Other sinonasal compartments | Pre-maxillary | Retro-maxillary | CNS |
| --- | --- | --- | --- | --- | --- | --- | --- | --- |
|  |  |  |  |  |  |  |  |  |
| Nasal cavity (37%) | 58 (100) | 9 (16) | 3 (5) | 2 (6) | 11 (19) | 1 (0.5) | 1 (0.5) | - |
| Maxillary sinus (34%) | 52 (100) | 10 (27) | 20 (38) | 7 (13) | 21 (40) | 12 (23) | 1 (0.5) | - |
| Ethmoid sinus (6%) | 12 (100) | - | 7 (38) | - | 6 (50) | - | - | 1 (58) |
| Sphenoid sinus (2%) | 4 (100) | - | 3 (75) | - | - | - | - | 1 (25) |
| Frontal sinus (2%) | 2 (100) | - | 1 (50) | - | 1 (50) | - | - | - |

Extension of sinonasal DLBCL to other adjacent anatomical regions, by primary site. Patients with multiple sites or unverifiable sinus involvement not included. Sum of percentages >100% due to the extension of tumor to multiple anatomical compartments. DLBCL: diffuse large B-cell lymphoma.
