# Supplementary material for: Sinonasal B‐cell lymphomas: A nationwide cohort study, with an emphasis on the prognosis and the recurrence pattern of primary diffuse large B‐cell lymphoma
Source: Hematol Oncol. 2022 Feb 6;40(2):160–71. doi: 10.1002/hon.2968 (PMC9303446; doi:10.1002/hon.2968)
Supplement: Supplementary file 6 — Table S3 [file HON-40-160-s007.docx]

Supplementary Table S3. Patient characteristics, other sinonasal lymphomas.

|  |  | FL n (%) | LPL n (%) | BL n (%) | EMZL n (%) | LGBCL n (%) |
| --- | --- | --- | --- | --- | --- | --- |
| **Total** |  | 4 (100) | 4 (100) | 3 (100) | 3 (100) | 2 (100) |
| % of all lymphomas |  | 1.9 | 1.9 | 1.5 | 1.5 | 1 |
| Male |  | 0 (0) | 4 (100) | 3 (100) | 2 (67) | 1 (50) |
| **Age at diagnosis** |  |  |  |  |  |  |
| Median [range], y |  | 66.7  [67.2–69.2] | 74.2 [66.6–80.0] | 72.8 [29.2–86.6] | 75.1  [71.0–76.0] | 55.7  [43.8–80.0] |
| **Disease group** |  |  |  |  |  |  |
| Primary, without lymph node involvement |  | 3 (75) | 1 (25) | - | - | - |
| Primary, with lymph node involvement |  | - | 1 (25) | 1 (33) | - | - |
| Secondary |  | 1 (25) | 2 (50) | 2 (67) | 3 (100) | 2 (100) |
| **Sinonasal region as presenting site** |  |  |  |  |  |  |
| Yes |  | 3 (75) | 2 (50) | 3 (100) | 2 (67) | 1 (50) |
| No |  | 1 (25) | 2 (50) | - | 1 (33) | - |
| Unknown |  | - | - | - | - | 1 (50) |
| **Elevated LDH** |  |  |  |  |  |  |
| No |  | 3 (75) | 2 (50) | 1 (33) | 3 (100) | 2 (100) |
| Yes |  | 1 (25) | - | 2 (67) | - | - |
| Unknown |  | - | 2 (50) | - | - | - |
| **Location** |  |  |  |  |  |  |
| Nasal cavity |  | 1 (25) | 2 (50) | 1 (33) | 1 (33) | 1 (50) |
| Maxillary sinus |  | 2 (50) | 1 (25) | 1 (33) | 1 (33) | 1 (50) |
| Ethmoid sinus |  | 1 (25) | - | - | - | - |
| Sphenoid sinus |  | - | - | - | - | - |
| Frontal sinus |  | - | - | - | - | - |
| Multiple |  | - | 1 (25) | 1 (33) | 1 (33) | - |
| **Laterality** |  |  |  |  |  |  |
| Unilateral |  | 4 (100) | 4 (100) | 2 (67) | 1 (33) | 2 (100) |
| Bilateral |  | - | - | 1 (33) | 1 (33) | - |
| Unknown |  | - | - | - | 1 (33) | - |
| **B symptoms** |  |  |  |  |  |  |
| No |  | 4 (100) | 3 (75) | 1 (33) | 3 (100) | 1 (50) |
| Yes |  | - | 1 (25) | 2 (67) | - | 1 (50) |
| **Ann Arbor stage** |  |  |  |  |  |  |
| IE |  | 3 (75) | 1 (25) | - | - | - |
| IIE |  | - | 1 (25) | 1 (33) | - | - |
| III |  | 1 (25) | - | - | 1 (33) | - |
| IV |  | - | 2 (50) | 2 (67) | 2 (67) | 2 (100) |
| **AJCC** |  |  |  |  |  |  |
| IE |  | 3 (75) | 1 (25) | - | - | - |
| IIE |  | - | 1 (25) | 1 (33) | - | - |
| IV |  | 1 (25) | 2 (50) | 2 (67) | 3 (100) | 2 (100) |
| **Performance status** |  |  |  |  |  |  |
| 0 |  | 4 (100) | - | 2 (67) | 3 (100) | 1 (50) |
| 1 |  | - | 3 (75) | - | - | 1 (50) |
| 2 |  | - | - | - | - | - |
| 3 |  | - | - | 1 (33) | - | - |
| 4 |  | - | - | - | - | - |
| Unknown |  | - | 1 (25) | - | - | - |
| **Relapse/progression of lymphoma** |  |  |  |  |  |  |
| No relapse or progression |  | 3 (75) | 1 (25) | 1 (33) | 1 (33) | 1 (50) |
| Relapse from primary |  | - | - | 1 (33) |  | - |
| Relapse from secondary |  | - | - | - | 1 (33) | 1 (50) |
| Progression of refractory lymphoma |  | 1 (25) | 2 (50) | 1 (33) | 1 (33) | - |
| Unknown |  | - | 1 (25) | - | - | - |
| **Response** |  |  |  |  |  |  |
| Complete response |  | 3 (75) | 1 (25) | 2 (67) | 2 (67) | 2 (100) |
| Partial response |  | - | - | 1 (33) | 1 (33) | - |
| Stable disease |  | 1 (25) | 2 (50) | - | - | - |
| Died before evaluation |  | - | 1 (25) | - | - | - |
| Unknown |  | - | - | - | - | - |
| **Status at last follow-up** |  |  |  |  |  |  |
| Alive with complete remission |  | - | - | 1 (25) | 1 (33) | - |
| Alive with disease |  | 1 (25) | 1 (25) | 2 (67) | - | 1 (50) |
| Dead from lymphoma |  | - | 1 (25) | - | 2 (67) | 1 (50) |
| Dead from other cause |  | 3 (75) | 2 (50) | - | - | - |
| **Median time to death from lymphoma** |  | NA | 0.29 [NA] | 4.26  [NA] | 3.54 [1.72–5.34] | 6.47  [NA] |

FL, follicular lymphoma; BL, Burkitt lymphoma; EMZL, extranodal marginal zone B-cell lymphoma; LGBCL, low-grade B-cell lymphoma; LPL, lymphoplasmacytic lymphoma.
