# Supplementary material for: Sinonasal B‐cell lymphomas: A nationwide cohort study, with an emphasis on the prognosis and the recurrence pattern of primary diffuse large B‐cell lymphoma
Source: Hematol Oncol. 2022 Feb 6;40(2):160–71. doi: 10.1002/hon.2968 (PMC9303446; doi:10.1002/hon.2968)
Supplement: Supplementary file 7 — Table S4 [file HON-40-160-s006.docx]

Supplementary Table S4. Initial treatment of sinonasal diffuse large B-cell lymphoma.

| **Stage** | IE | IIE | III | IV | NA |
| --- | --- | --- | --- | --- | --- |
| Number | 107 (100) | 24 (100) | 5 (100) | 20 (100) | 7 (100) |
| **Chemotherapy regimen** |  |  |  |  |  |
| CHOP | 66 (61) | 12 (50) | 5 (100) | 11 (51) | - |
| CEOP | 3 (3) | 2 (8) | - | - | - |
| CHOEP | 3 (3) | - | - | 1 (5) | - |
| COP | 4 (4) | 1 (4) | - | 2 (10) | - |
| CNOP | 6 (6) | 1 (4) | - | 1 (5) | - |
| Other | 5 (5) | 2 (8) | - | 1 (5) | - |
| **Treatment combination** |  |  |  |  |  |
| Chemotherapy  (mono) | 17 (16) | 3 (13) | 1 (20) | 4 (20) | - |
| Chemotherapy and radiotherapy | 24 (22) | 1 (4) | 1 (20) | 4 (20) | - |
| Immunochemotherapy | 18 (17) | 8 (33) | 3 (60) | 4 (20) | - |
| Immunochemotherapy and radiotherapy | 28 (26) | 6 (25) | - | 4 (20) | - |
| **Non-chemotherapy treatment** |  |  |  |  |  |
| Antimetabolites and/or alkylating chemotherapy | 1 (1) | - | - | 1 (5) | - |
| Radiotherapy  (mono) | 12 (11) | 3 (13) | - | - | - |
| Immunotherapy (mono) | 1 (1) | - | - | - | - |
| Immunotherapy and radiotherapy | 2 (2) | - | - | - | - |
| CNS prophylaxis* | 34 (32) | 8 (33) | 2 (40) | 7 (35) | - |
| ABMT* | 3 (3) | 1 (4) | - | - | - |
| No treatment | 1 (1) | 2 (8) | - | 2 (10) | 2 (29) |
| Unknown | 3 (3) | 1 (4) | - | 1 (5) | 5 (71) |

Table 3. Detailed discription of treatment received by patients with diffuse large B-cell lymphoma; high-grade B-cell lymphoma with or without double-hit is not included. CHOP: cyclophosphamide, hydroxydaunomycin (doxorubicin), Oncovin® (vincristine), and prednisolone. CHOEP: CHOP + etoposide. CVP/COP: cyclophosphamide, Oncovin®/vincristine, and prednisolone. CNOP: CVP/COP + mitoxantrone (Novantrone). ABMT: autologous bone marrow transplant. Other regimes included MIME (methyl-gag, ifosfamide, methotrexate, and etoposide), DHAP (dexamethasone, high-dose Ara-C, and cisplatin [Platinol®]; ICE (ifosfamide, carboplatin, and etoposide); and VIM (ifosfamide, mitoxantrone, and etoposide). *In addition.
